# A Triple Threat: Immune Checkpoint Inhibitor-Induced Diabetic Ketoacidosis, Myocarditis, and Adrenal Insufficiency in a Patient With Metastatic Melanoma

**DOI:** 10.7759/cureus.94583

**Published:** 2025-10-14

**Authors:** Aamir Mohamed Nur, Mohammed Aamir

**Affiliations:** 1 Haematology, University Hospitals of Leicester NHS Trust, Leicester, GBR

**Keywords:** diabetic keto acidosis, immune-checkpoint inhibitors, immune-related adverse effects, immune therapy mediated myocarditis, immunotherapy induced dka, ipilimumab nivolumab

## Abstract

Immune checkpoint inhibitors (ICIs), such as ipilimumab and nivolumab, have revolutionized cancer therapy, particularly in advanced melanoma. By enhancing T-cell-mediated anti-tumor activity, these agents have significantly improved survival in metastatic disease. However, this immune activation can result in a range of immune-related adverse events (irAEs), which may affect virtually any organ system, sometimes with overlapping or atypical presentations. Endocrine irAEs, such as hypophysitis, thyroid dysfunction, and adrenalitis, are relatively well-described. Less commonly, ICIs can precipitate insulin-dependent diabetes mellitus or autoimmune myocarditis - both of which are rare but potentially life-threatening. These complications may present non-specifically, such as with fatigue or malaise, and are often under-recognized. This case highlights the importance of maintaining a high index of suspicion for multiple concurrent irAEs in patients on immunotherapy, even in the absence of overt clinical signs. Early recognition and prompt immunosuppressive therapy can significantly reduce morbidity and improve outcomes.

## Introduction

A 58-year-old woman with metastatic melanoma, receiving combination ipilimumab and nivolumab, presented with fatigue and was diagnosed with diabetic ketoacidosis (DKA). Laboratory findings revealed hyperglycemia, ketonemia, low C-peptide, and positive glutamic acid decarboxylase (GAD) antibodies, confirming immune checkpoint inhibitor (ICI)-induced autoimmune diabetes, a recognized immune-related adverse event (irAE) associated with ICIs [1]. The onset of autoimmune diabetes in the setting of immunotherapy highlights the importance of considering this diagnosis in patients presenting with metabolic abnormalities, even without a prior history of diabetes [2]. Additionally, the patient’s low morning cortisol level indicated adrenal insufficiency, consistent with ICI-induced adrenalitis, a known complication of immunotherapy [3]. Imaging ruled out adrenal hemorrhage, and she was started on high-dose corticosteroids, which is the standard management for ICI-induced adrenalitis. This diagnosis was made following the exclusion of other causes, including metastatic disease [4]. Markedly elevated pro-B-type natriuretic peptide (pro-BNP) raised concern for ICI-induced myocarditis, despite the patient being clinically stable with normal echocardiogram findings. Myocarditis related to ICIs can present with subtle symptoms and elevated cardiac biomarkers, such as pro-BNP and troponins, even when echocardiograms are unremarkable [5]. The patient’s pro-BNP level decreased dramatically after initiating high-dose methylprednisolone, supporting the diagnosis of myocarditis. The patient was treated with a multidisciplinary approach, focusing on the three main toxicities: DKA, adrenal insufficiency, and myocarditis. Following stabilization, she was transitioned to subcutaneous insulin therapy (Lantus and Trurapi) for autoimmune diabetes. Her adrenal insufficiency was managed with oral prednisolone and a tapering plan. Multidisciplinary care involving endocrinology and cardiology was crucial to her recovery and ongoing management, underscoring the importance of timely identification and treatment of irAEs in patients receiving ICIs [6].

## Case presentation

A 58-year-old woman with a past medical history of hypertension, chronic obstructive pulmonary disease (COPD), and hypothyroidism was undergoing palliative immunotherapy for metastatic melanoma. She had completed three cycles of combination ipilimumab (3 mg/kg) and nivolumab (1 mg/kg). She presented to the department with generalized fatigue of several days’ duration. She denied fever, chest pain, dyspnoea, nausea, vomiting, or any other infectious symptoms. Physical examination was unremarkable apart from mild lethargy. Vital signs were stable. Initial investigations are shown in Table 1.

**Table 1:** Laboratory investigations VBG: venous blood gas; pro-BNP: pro-B-type natriuretic peptide; GAD: glutamic acid decarboxylase; CRP: C-reactive protein; DKA: diabetic ketoacidosis

Investigation	Result	Clinical Relevance	Reference Ranges
VBG	pH: 7.254, HCO_3_: 14.4 mmol/L	Confirms metabolic acidosis (DKA)	pH: 7.32-7.43, HCO_3_: 20-28 mmol/L
Ketones	6.1 mmol/L	Diagnostic for DKA	<0.6 mmol/L
Blood glucose	25.5 mmol/L	Hyperglycemia confirming DKA	3.6-5.3 mmol/L
Cortisol	71 nmol/L	Suggests adrenal insufficiency	145-619 nmol/L
Pro-BNP	1118 pg/mL	Suggests myocarditis	20-200 pg/mL
C-peptide	13 pmol/L	Low insulin production → autoimmune diabetes	268-1275 pmol/L
GAD antibodies	75 IU/mL	Confirms autoimmune diabetes	0-9 IU/mL
CRP	14 mg/L	Rules out infection	0-10 mg/L

**Figure 1:**
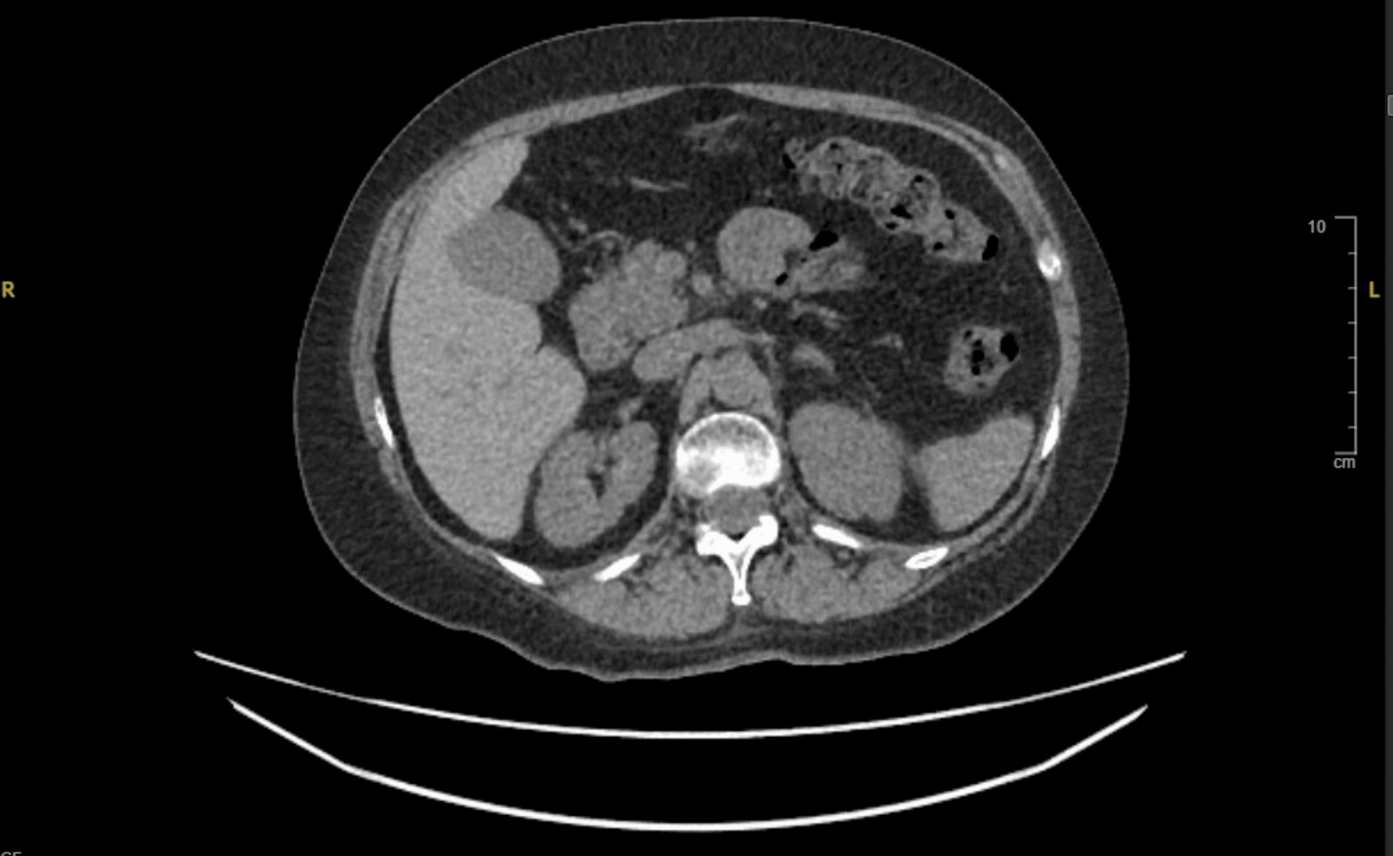
Computed tomography (CT) of the abdomen and pelvis, showing no evidence of adrenal hemorrhage.

The patient had no prior diagnosis of diabetes. The constellation of findings confirmed DKA, and she was immediately started on standard DKA protocol with intravenous fluids and insulin infusion. Given the low serum cortisol, endocrinology was consulted. They attributed the result to ICI-induced adrenal insufficiency, likely primary adrenalitis, a known irAE. A CT abdomen/pelvis was performed to rule out adrenal hemorrhage, which was not identified (as shown in Figure 1). To investigate the cause of her fatigue further, a pro-BNP level was taken, returning markedly elevated at 1118 pg/mL. There was no history of cardiac disease. Although she was hemodynamically stable and without overt cardiac symptoms, these findings raised suspicion of ICI-induced myocarditis, another rare but serious irAE. The patient was initiated on high-dose intravenous methylprednisolone (4 mg/kg/day). This led to a rapid reduction in pro-BNP levels to 115pg/mL, providing biochemical support for the myocarditis diagnosis. A transthoracic echocardiogram (TTE) was performed, showing normal left ventricular (LV) size and systolic function (visual left ventricular ejection fraction (LVEF) >54%) with concentric remodeling. The right ventricle (RV) demonstrated normal size and longitudinal function. A tiny pericardial effusion, not measurable in diastole, was noted and may represent adipose tissue or fibrinous material. Diastolic function could not be assessed.

Endocrinology advised starting Lantus (insulin glargine) 12 units daily and Trurapi (rapid-acting insulin) three times daily with meals following DKA resolution. An autoimmune diabetes screen revealed the following result: C-peptide: 13 pmol/L (low, consistent with insulin deficiency) and a GAD antibody: 75 IU/mL (strongly positive). These findings confirmed ICI-induced autoimmune diabetes. The patient was transitioned to subcutaneous insulin therapy and began a weaning dose of oral prednisolone as her clinical condition stabilized. The patient’s primary symptom of generalized fatigue, in the context of immunotherapy and stable vital signs, required a broad differential diagnosis. Her known comorbidities (COPD, hypertension, and hypothyroidism) and metastatic melanoma further complicated the clinical picture. A systematic stepwise diagnostic approach was undertaken to explore both common and immune-related causes.

The patient presented with DKA, as demonstrated by significantly elevated serum glucose (25.5 mmol/L), high ketones (6.1 mmol/L), metabolic acidosis (pH 7.254, bicarbonate 14.4 mmol/L), and a raised anion gap. Given the rapid onset of symptoms and the lack of any prior diabetes history, further exploration into the underlying cause of the diabetes was prompted. While type 2 diabetes with ketosis was initially considered, it was considered unlikely due to the absence of prior hyperglycemia and the rapid progression of symptoms. Exclusion of other potential causes, such as pancreatic metastases leading to islet destruction or steroid-induced diabetes, was achieved through imaging and the timing of symptom onset. The presence of low C-peptide and positive GAD antibodies strongly suggested an autoimmune etiology, confirming the diagnosis of autoimmune diabetes. The clinical presentation, coupled with the known association of ICIs with autoimmune diabetes, pointed towards ICI-induced type 1 diabetes, a known irAE.

In addition to the DKA, the patient's adrenal insufficiency was highlighted by a low morning cortisol level of 71 nmol/L, prompting a thorough evaluation for potential causes. The most likely diagnosis was ICI-induced primary adrenalitis, given the temporal association with immunotherapy and the absence of exogenous steroid use. Other possibilities, such as pituitary or hypothalamic involvement (hypophysitis), were considered but deemed less likely due to the absence of key features like headaches, visual changes, or deficiencies in other pituitary hormones. Further endocrine workup, including adrenocorticotropic hormone (ACTH), thyroid-stimulating hormone (TSH), and luteinizing hormone/follicle-stimulating hormone (LH/FSH) testing, could help differentiate between central and peripheral causes of adrenal insufficiency. Imaging studies, including CT scans, excluded structural causes such as adrenal metastases, hemorrhage, or tumor infiltration, while the absence of signs of infection, fever, or hemodynamic instability excluded sepsis-induced adrenal dysfunction. These findings strongly supported a diagnosis of ICI-induced primary adrenal insufficiency, which was particularly likely given the patient's concurrent autoimmune diabetes. The cardiac evaluation revealed a markedly elevated pro-BNP (1118 pg/mL), raising suspicion for myocarditis, particularly given the patient’s immunotherapy background. Although echocardiography showed preserved systolic function and only a small pericardial effusion, the absence of chest pain, dyspnea, or hypotension necessitated consideration of alternative diagnoses. Other potential causes, including heart failure, pulmonary embolism, sepsis-related cardiomyopathy, and renal impairment, were considered but excluded based on clinical signs and imaging results. Heart failure was considered unlikely because the patient was euvolemic, and her LVEF was preserved. Similarly, pulmonary embolism was ruled out due to the lack of tachycardia, hypoxia, or pleuritic symptoms, and sepsis was unlikely because there were no signs of systemic infection, such as fever, leukocytosis, or hypotension, and the patient's condition remained stable with serial observations, ruling out sepsis as a contributing factor. Although myocarditis can present with subtle findings, the significant drop in pro-BNP after corticosteroid therapy strongly supported the diagnosis of ICI-induced myocarditis. A definitive diagnosis would require further tests, such as cardiac MRI or biopsy, but the clinical and biochemical response to steroids, combined with the exclusion of other causes, pointed to myocarditis as the most likely etiology. The patient’s history of hypothyroidism raised concern for possible decompensated thyroid dysfunction contributing to her fatigue, but thyroid function tests, though not explicitly detailed, were assumed to be checked and showed no significant derangement. Moreover, there were no signs of myxedema (such as bradycardia, hypothermia, or hyponatremia), further ruling out this as a primary cause.

The possibility of underlying malignancy progression, particularly metastatic melanoma, was considered in view of the patient's history of cancer and symptoms such as fatigue and adrenal dysfunction. However, imaging studies, including a CT scan, revealed no acute oncologic complications, such as adrenal metastases, hepatic failure, or intracranial disease, making progression of the underlying malignancy unlikely. COPD exacerbation was another potential cause of fatigue, given the patient's age. However, there were no respiratory symptoms, no signs of hypoxia or wheezing, and CO_2_ retention was ruled out through a venous blood gas (VBG). Lastly, no clinically significant electrolyte abnormalities were identified, and a medication review revealed no changes in the patient's treatment regimen that could explain her fatigue or the development of DKA. The combination of these findings helped narrow down the most likely diagnoses, with the working hypothesis being ICI-induced autoimmune diabetes, adrenal insufficiency, and myocarditis. Other potential causes were carefully considered and excluded.

The final diagnoses of ICI-induced autoimmune diabetes, primary adrenalitis, and myocarditis were established after careful exclusion of other potential endocrinological, infectious, oncological, and cardiovascular causes. The simultaneous occurrence of these irAEs emphasized the need for heightened clinical suspicion and a coordinated multidisciplinary approach in managing patients on ICIs. The patient was initially treated according to standard DKA protocols, which included IV fluids, insulin infusion, and potassium replacement, with normalization of blood glucose, ketones, and acid-base status within 48 hours. Given the absence of prior diabetes, autoimmune diabetes was suspected. For adrenal insufficiency, a cortisol level of 71 nmol/L prompted an urgent referral to endocrinology, and stress-dose IV methylprednisolone (4 mg/kg/day) was started while imaging ruled out adrenal hemorrhage. The patient was transitioned to oral prednisolone with a tapering plan upon discharge. Suspected ICI-induced myocarditis was treated with high-dose methylprednisolone, which led to a significant drop in pro-BNP levels (from 1118 pg/mL to 115 pg/mL), with clinical stability and preserved systolic function on TTE, indicating subclinical myocarditis. Long-term management of diabetes included the initiation of subcutaneous insulin (Lantus 12 units daily and Trurapi three times daily with meals) after autoimmune diabetes was confirmed via low C-peptide levels and positive GAD antibodies. The diagnosis of ICI-induced autoimmune type 1 diabetes was confirmed, and the patient was referred to community endocrinology for ongoing diabetes management. After completing the steroid taper, she remained clinically stable and was discharged home with follow-up appointments scheduled in both endocrinology and oncology.

## Discussion

This case highlights a rare but clinically significant constellation of multiple irAEs in a patient undergoing combination ICI therapy. The simultaneous occurrence of immune-mediated type 1 diabetes mellitus, adrenalitis, and myocarditis - all within a single presentation - underscores the systemic immune activation that ICIs can provoke and the importance of early recognition and multidisciplinary management. ICIs such as ipilimumab (anti-CTLA-4) and nivolumab (anti-PD-1) act by reversing T-cell inhibition, thereby enhancing anti-tumor immunity. However, this immune activation can become dysregulated, leading to T-cell-mediated destruction of healthy tissues - particularly in endocrine organs and the myocardium [7]. In the endocrine system, activated cytotoxic T lymphocytes can target pancreatic islet cells (leading to autoimmune diabetes) or adrenal cortex (resulting in adrenalitis) [8]. Myocarditis, although rare (<1% incidence), is among the most lethal ICI-related toxicities, often occurring early in the treatment course and potentially without overt cardiovascular symptoms [9]. ICI-induced diabetes is an emerging clinical entity, typically resembling type 1 diabetes, with rapid onset, insulin deficiency, and frequent presentation as DKA [10]. The detection of positive GAD antibodies and low C-peptide levels in this patient confirmed the autoimmune nature of her diabetes. Unlike classical type 1 diabetes, ICI-induced diabetes may occur in older patients and lacks the typical preclinical phase [11]. Routine screening for hyperglycemia is not currently mandated in major guidelines, but clinicians are encouraged to monitor glucose levels during therapy, particularly when nonspecific symptoms like fatigue occur [12]. Adrenal insufficiency secondary to ICIs may be primary (adrenalitis) or secondary (hypophysitis). In this case, the isolated low cortisol with an unremarkable pituitary profile and normal imaging excluded central causes. The absence of adrenal hemorrhage or metastases on CT further supported a diagnosis of primary autoimmune adrenalitis, consistent with published irAE patterns [13]. Importantly, adrenalitis may present insidiously with non-specific symptoms such as lethargy or malaise, and delayed diagnosis can result in adrenal crisis. Guidelines from the European Society for Medical Oncology (ESMO) recommend baseline and periodic monitoring of cortisol and ACTH in patients receiving ICIs, especially when symptoms arise [14]. Myocarditis from ICIs is rare but can be fatal, with a mortality rate of up to 50% [15]. The diagnosis is challenging due to its frequently subclinical presentation. In our patient, the isolated elevation in pro-BNP prompted further evaluation despite the absence of cardiac symptoms. The TTE was largely unremarkable, but the rapid biochemical response to high-dose corticosteroids supported an inflammatory etiology. Cardiac MRI or endomyocardial biopsy is considered the gold standard for confirmation, though not always feasible. The American Society of Clinical Oncology (ASCO) guidelines recommend initiating empiric corticosteroids (1-2 mg/kg/day prednisone or equivalent) when myocarditis is suspected, even before definitive imaging is performed [16].

While individual irAEs are increasingly reported, concurrent presentation of multiple endocrinopathies and myocarditis is rare. Table 2 summarizes a selection of similar cases from the literature.

**Table 2:** Discussion of similar cases ICI: immune checkpoint inhibitor; irAEs: immune-related adverse events; DKA: diabetic ketoacidosis; pro-BNP: pro-B-type natriuretic peptide; anti-PD-1: anti-programmed cell death protein 1; anti-CTLA-4: anti-cytotoxic T-lymphocyte-associated protein 4

Author (Year)	ICI Regimen	irAEs	Presentation	Outcome
Stamatouli et al. (2018) [10]	Anti-PD-1	Autoimmune diabetes	DKA, hyperglycemia	Insulin-dependent
Mahmood et al. (2018) [15]	Anti-CTLA-4 + PD-1	Myocarditis	Elevated troponin, fatigue	High mortality
Quandt et al. (2020) [8]	Anti-PD-1	Adrenalitis + diabetes	Hypoglycemia fatigue	Corticosteroids insulin
Present case	Anti-CTLA-4 + PD-1	Diabetes, adrenalitis, myocarditis	Fatigue, DKA, ↑pro-BNP	Stabilized on insulin and steroids

This case underscores the protean and potentially concurrent manifestations of ICI-related toxicity. Fatigue, while often benign, was the sentinel symptom of three serious irAEs. Prompt recognition, exclusion of alternative causes, and timely initiation of immunosuppression led to clinical stabilization. Greater awareness, routine monitoring, and adherence to evolving irAE management guidelines can mitigate the morbidity and mortality associated with these life-threatening complications.

## Conclusions

Non-specific symptoms like fatigue in patients undergoing ICI therapy should prompt a broad differential diagnosis, as they may signal the presence of multiple simultaneous irAEs. Even in the absence of classic or overt clinical signs, ICI-induced endocrinopathies, such as adrenal insufficiency or autoimmune diabetes, and myocarditis should always be considered, particularly during the early phases of treatment when the risk of these complications is heightened. A low threshold for initiating endocrine and cardiac workups, including tests for cortisol, glucose, BNP, and troponins, can significantly aid in the early identification of these irAEs, allowing for more timely interventions. Furthermore, a multidisciplinary management approach that involves specialists in endocrinology, cardiology, and oncology is essential for optimizing patient outcomes and preventing further complications. Despite the growing recognition of these toxicities, there is a clear need for more defined guidelines on screening, monitoring, and surveillance, especially for high-risk patients, such as those receiving combination immunotherapy regimens. Tailored surveillance protocols could ensure earlier detection, more efficient management, and improved long-term care for these patients, ultimately enhancing the benefits of immunotherapy while minimizing the risks of irAEs.
